# Cross-reaction between mouse and rat immunoglobulin G: does it matter in sandwich ELISA?

**DOI:** 10.1186/s43141-021-00222-2

**Published:** 2021-08-17

**Authors:** Rola Nadeem, Ahmed B. Barakat, Mahmoud M. Bahgat

**Affiliations:** 1 Department of Therapeutic Chemistry, Division of Pharmaceutical and Drug Industries Research, the National Research Center, Cairo, Egypt; 2 Research Group Immune- and Bio-markers for Infection, The Center of Excellence for Advanced Sciences, the National Research Center, El Buhooth Street (Formerly El Tahrir), Dokki, Cairo, 12622 Egypt; 3 Department of Microbiology, Faculty of Science, Ain Shams University, Cairo, Egypt

**Keywords:** Sandwich ELISA, Cross-reaction, Anti-mouse IgG, Anti-rat IgG, Capture-antibody, Detection-antibody

## Abstract

**Background:**

Sandwich ELISA is an ideal antigen detection and quantification assay. Recently, it was used as the basic concept for high technology diagnostics. The specificity of the assay depends on the exclusion of detection cross-reactivity which arises from using two antibodies developed in different species. Since mice and rats are the common laboratory animals used to develop antigen specific antibodies. Therefore, the questions we addressed here were (1) can one use antigen-specific antibodies raised in mice and rats in the same assay to specifically detect/quantify antigens? and (2) which antibodies of the two rodents should be placed for capturing and for detection in the antigen-detection sandwich?

**Results:**

Direct ELISA assay was used to assess for the specific reaction of the HRP-conjugated antibody to the target serum. First reaction was to compare between either conjugate anti-rat IgG (homologous) or anti-mouse IgG (heterologous) for the detection of rat sera IgG. Following the dilution factor optimization, the O.D. were 0.744±0.051 and 0.604±0.05, respectively (*p=* .004). The difference in mean O.D. of 0.14 reflected an unaccepted non-specific reaction. The second reaction was to compare between either conjugate anti-mouse IgG (homologous) and anti-rat IgG (heterologous) for the detection of mouse sera IgG. The recorded O.D. were 0.9414±0.14 and 0.317 ±0.141, respectively (*p=* .0002). The improved difference in mean O.D. of 0.624 reflecting a minimized cross-reaction.

**Conclusions:**

Our results suggest that it is possible to use both Swiss albino mice and albino rats in a single sandwich ELISA, given that the captured antibody species to be from the Swiss albino mice and the detection antibody to be from the albino rat. The described working details are limited to the source of the antibodies used in the study. However, the approach stresses on the importance of such optimization steps before making any interpretations based on the antigen detection. To our knowledge, this study is the first to cover the optimal order of the capturing and the detection antibodies in a sandwich ELISA assay. In addition to addressing the possible interfering cross-reactivity that result from using mouse and rat serum antibodies in a single assay.

**Graphical abstract:**

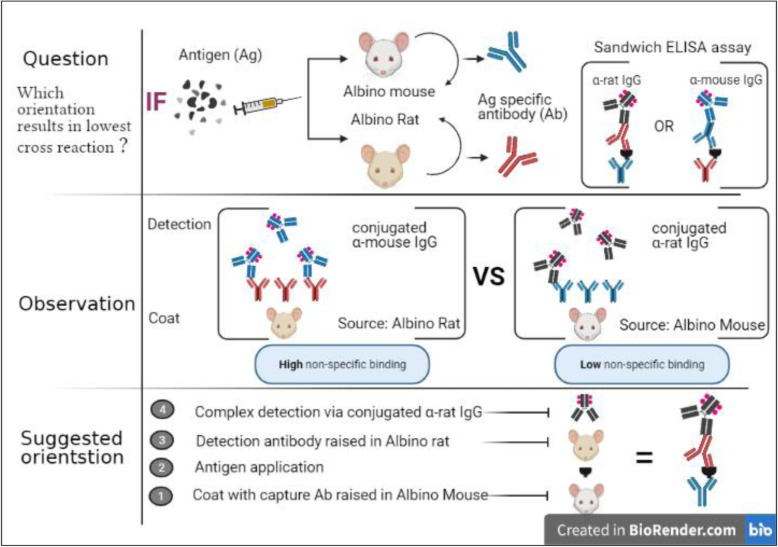

## Background

Immunoassays are used to quantify biomolecules (analytes) using specific antibodies produced against these particular molecules [1]. Since 1978, when ELISA was first described [2], it became the gold standard antigen-antibody detection assay in basic research of which some assays were further optimized for diagnostic purposes and became commercially available. More recent point-of-care diagnostic technologies such as smart cards [3] and lab-on-chip technology [4] were also based on the same principles of ELISA. Among the different types of ELISA, sandwich ELISA is considered a more sensitive and robust assay and tends to be the most commonly used type. Sandwich ELISA targets one antigen using two different antigenic sites detected by two different antibodies in the same assay. The first is known as “the capturing antibody” that is bound to the solid phase. This antibody captures the analyte from the sample then another antibody known as “the detection antibody” is used to detect the antigen bound—now sandwiched [1].

Diagnostic studies rely on sandwich ELISA as a key assay in many fields. It is used in the detection of bacterial [5], parasitic [6], fungal [7], and viral infections in both human and animals [8] as well as in botany research to detect plant viral disease [9]. In addition, it is also used in biomarker profiling [10, 11] and biomarker-based diagnostic assays for cancer diagnosis [12], diagnosis of autoimmune diseases [13], and Alzheimer’s [14]. Moreover, it has applications in vaccine development [15] and conjugate vaccine assessment [16]. Also, it is of use in discrimination of allergic cultivates for human [17] and even as a basic bio-approach in engineering as in early-diagnostic biosensors [18].

Sandwich ELISA, despite being a conventional assay, was in many cases further developed into time saving diagnostic kits. One successful example is the immunochromatographic card (ICT) for qualitative detection of circulating filariasis, *Wuchereria bancrofti*, antigen [19]. The test card was further optimized into semi-quantitative card that does not only detect but also helps in grading the degree of infection [20]. Now this rapid test-card is used as a point-of-care diagnostic assay to detect and follow-up patient’s response to therapy [21]. Despite being a simple and direct principle, it remains a key assay due to both the sensitivity and specificity of the detection which emphasizes the importance of the development and optimization of the reaction conditions for various applications.

Sandwich ELISA development and optimization includes the selection of matrices, buffers, and detective means but most importantly the choice of a matching capturing and detection antibodies and the orientation of the binding assembly [22]. This double antibody detection assay should be using two non-overlapping, non-interfering antigen-specific antibodies with a minimal or completely absent cross-reactivity. This raises not only a concern about the quality and specificity of the antibody, but also the source host where the antibodies were raised. For commercially available basic antibodies, this can be solved by choosing the host of the antibody from a panel of available labeled and unlabeled antibodies. On the other hand, in case of studying a specific agent analyte, the antibodies should be raised in laboratory animals.

Mice and rats remain the top chosen laboratory animals due to their feasible manipulation and breeding conditions, low cost, and the uncomplicated ethical approval. But it is well known that the two species directly relate phylogenetically to one another [23]. This raises the questions: to which extent the chosen mouse and rat species to raise anti-sera are immunologically related? And to which extent the developed antibodies in both models might cross-react with one another if used for capturing and detection in a sandwich ELISA?

Although these questions sound very trivial, and are frequently asked, we carefully searched the available literature and found no clear experimental answers for both. What increased the confusion was the commercial availability of antibodies that cross-react and detect both mice and rat immunoglobulins, while there is also both polyclonal rat-anti-mouse and mouse-anti-rat antibodies as matched pairs for rat and mouse-based sandwich ELISA without any hint or instructions about the extent of cross-reaction among both.

Therefore, the present work evaluates both anti-mouse IgG and anti-rat IgG for their possible cross-reactivity in the specific and non-specific detection of mouse and rat serum IgG. This evaluation aims to guide on the best species-serum order that allows minimal cross-reaction when establishing a sandwich ELISA.

## Methods

### Animals

Sera were collected from 5 female Swiss albino mice and 5 female albino rats of age 6 weeks. The average body weight was 29 and 120 g, respectively. Single blood sample was collected from the orbital venous plexus of each animal individually. The animal was anesthetized using diethyl ether vapor discontinuous sniffs till numbness to reduce awareness of pain. The animal was scuffed with thumb and forefinger and the skin around the eye is pulled taut. A heparinized capillary tube was inserted into the medial canthus of the eye. After sample collection, eyes were wiped with sterile cotton and bleeding was stopped by gentle finger pressure. Animals were checked for normal vital signs (breath, movement, feeding) and possible lesions for 30 min post collection procedure. Sera were separated by centrifugation at 10,000×*g*, divided into multiple aliquots, and frozen at −80 °C till being used.

### Method of direct ELISA assay

All dilution combinations were done in quadruplicates and all steps were incubated at 37 °C.

*Steps*:

- Microtiter plate (SPL life sciences, Korea) was coated with 50 μl with each individual animal serum diluted in carbonate/ bicarbonate coating buffer, pH= 9.6 for 2.5 h.
  Each individual animal serum was used as 1:500 and 1:1000 dilutions.
- Wells were washed 4 times using 300 μl PBS-0.05%-Tween-20 (PBST; pH= 7).
- Wells were blocked with 150 μl of blocking buffer (PBST-5%FBS) for 2 h.
- Wells were washed 4 times using 300 μl PBST.
- 50 μl of HRP-conjugate antibody was applied to its corresponding well for 1.5 h.
  Each of HRP-anti-mouse IgG and HRP-anti-rat IgG antibodies were used as 1:1000 and 1:2000 diluted in blocking buffer
- Wells were washed 4 times using 300 μl PBST.
- 50 μl of O-phenylenediamine (Sigma-Aldrich; Germany) in citrate substrate buffer (pH= 5) was added to the wells and monitored for color development
- Reaction was stopped using 50 μl of 2M H_2_SO_4_ to each well.
- Optical density (O.D., absorbance) was recorded using a microtiter plate reader (Tecan, Switzerland) at λ_max_ 490 nm using a 620 nm filter as a reference wavelength.

### Statistical analysis

Statistical significance of difference was calculated using the paired two-sample Student’s *t*-test (*p* value ≤ .05). For the calculation of the threshold value of signal to noise reaction of the conjugate antibody, a sample size of 5 of each animal serum is accepted. This was in reference to a previous study showing that for *N*=5 the type I error rate reach a nominal level of 5% [24]. Results were expressed as mean ± S.D. Statistical analysis and plots were done using the Microsoft Office Excel version 2016 software.

## Results

### Cross-reaction of HRP-anti-mouse-IgG in the detection of rat serum IgG

**Fig. 1:**
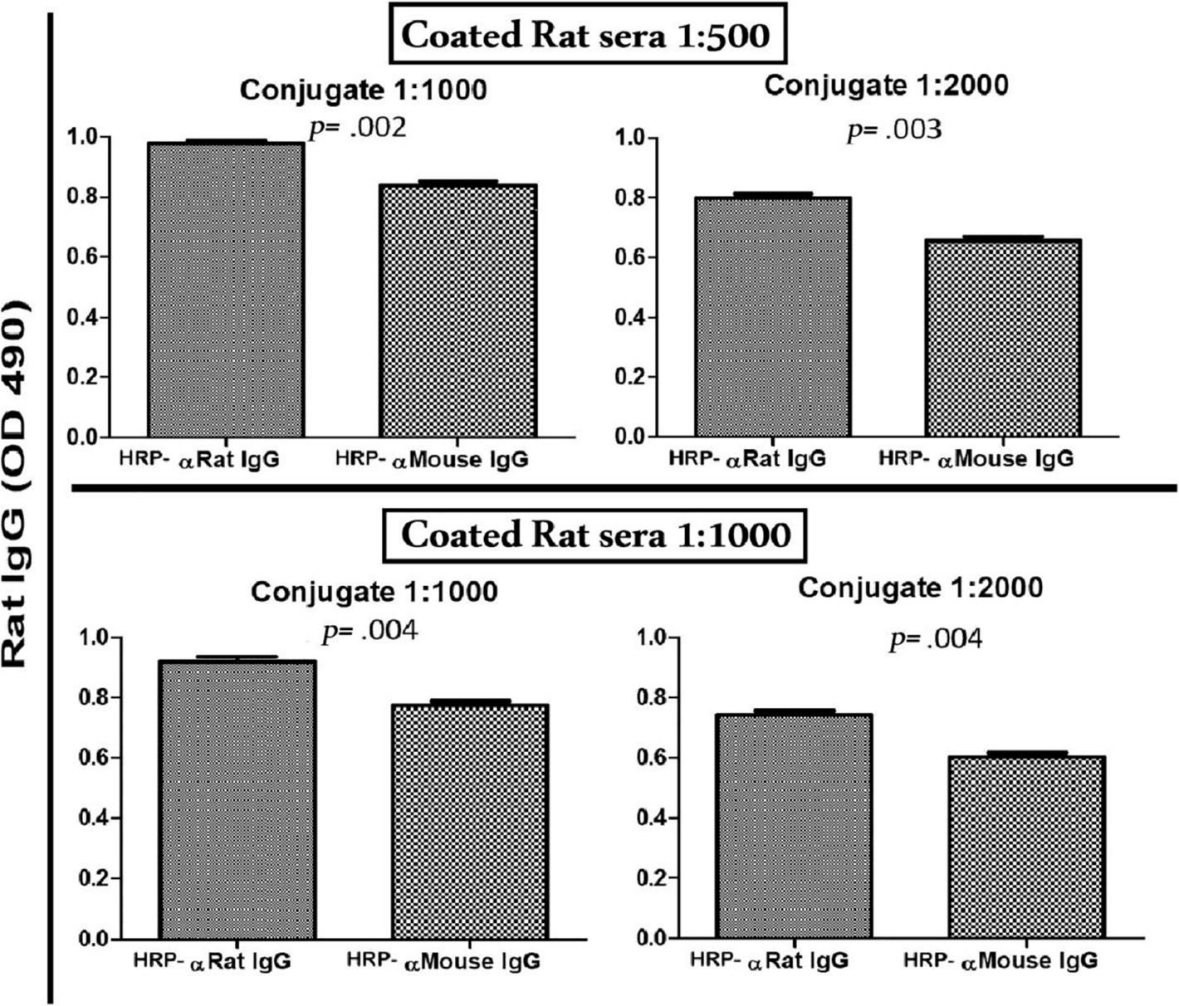
Reaction between individual rat serum (represented as mean O.D.) and both HRP-conjugated anti-rat IgG and HRP-conjugated anti-mouse IgG: Rat sera (*n* = 5) were used at dilutions 1:500 and 1:1000 and both conjugated antibodies were used at dilutions 1:1000 and 1:2000. Using Student’s *t*-test, results at different conditions showed statistically significant differences between reactivities with the homologous compared to the heterologous sera. However, the cross-reaction between rat sera and HRP-anti mouse-IgG remained unexpectedly and unacceptably high upon increasing both sera dilution to 1:1000 and conjugate dilution to 1:2000 (levels of reaction with anti-rat IgG and anti-mouse IgG were 0.744 ±0.051 and 0.604 ±0.05, respectively, as Δ mean O.D. = 0.14; *p=* .004)

Direct ELISA assay was used to compare between HRP-conjugated antibody of both anti-rat IgG (homologous) and anti-mouse IgG (heterologous) for the detection of rat sera IgG. The numerical results are shown in Table 1 for rat serum coat section and plotted in Fig. 1. Results revealed high degree of cross-reaction of HRP-anti-mouse-IgG to the rat serum IgG. The reaction of anti-mouse IgG that should have been non-specific, nearly resembled the specific conjugated anti-rat IgG antibody detection for the rat serum IgG. The dilution factor optimization started as rat serum coating the plates at dilution of 1:500 reacting with both conjugated antibodies at dilution 1:1000. The reaction with both homologous and heterologous antibodies were 0.977±0.02 and 0.837±0.05, respectively (*p=* .002), with a difference in mean (Δ mean O.D) of 0.14. This low difference could not be improved even by increasing the dilution factor of both rat serum to 1:1000 and conjugated antibody to 1:2000. The latter homologous and heterologous reactions were 0.744±0.051 and 0.604±0.05, respectively (*p=* .004) with a Δ mean O.D. = 0.14.

**Table 1:** Numerical O.D. values recorded for the reaction between coated mouse and rat serum with HRP-conjugated anti-IgG antibodies, showing working dilutions, respective O.D. and calculated **∆** O.D

Coated serum	Dilution factor	HRP-conjugated antibody	Dilution factor	O.D. ± SD	∆ O.D. (Homologous-heterologous serum test)
Rat serum	1:500	Anti-rat IgG	1:1000	0.977 ± 0.02	0.14 ± 0.03
		Anti-mouse IgG		0.837 ± 0.05	
		Anti-rat IgG	1:2000	0.798 ± 0.03	0.14 ±0.04
		Anti-mouse IgG		0.654 ± 0.06	
	1:1000	Anti-rat IgG	1:1000	0.918 ± 0.06	0.14 ± 0.05
		Anti-mouse IgG		0.776 ± 0.04	
		Anti-rat IgG	1:2000	0.744 ± 0.05	0.14 ±0.05
		Anti-mouse IgG		0.604 ± 0.05	
Mouse serum	1:500	Anti-mouse IgG	1:1000	1.094 ± 0.1	0.522 ± 0.16
		Anti-rat IgG		0.571 ± 0.23	
		Anti-mouse IgG	1:2000	1.015 ± 0.06	0.618 ± 0.13
		Anti-rat IgG		0.397 ± 0.2	
	1:1000	Anti-mouse IgG	1:1000	1.002 ± 0.16	0.539 ± 0.16
		Anti-rat IgG		0.463 ± 0.17	
		Anti-mouse IgG	1:2000	0.941 ± 0.14	0.624 ± 0.14
		Anti-rat IgG		0.317 ± 0.14

### Cross-reaction of HRP-anti-rat-IgG in the detection of mouse serum IgG

**Fig. 2:**
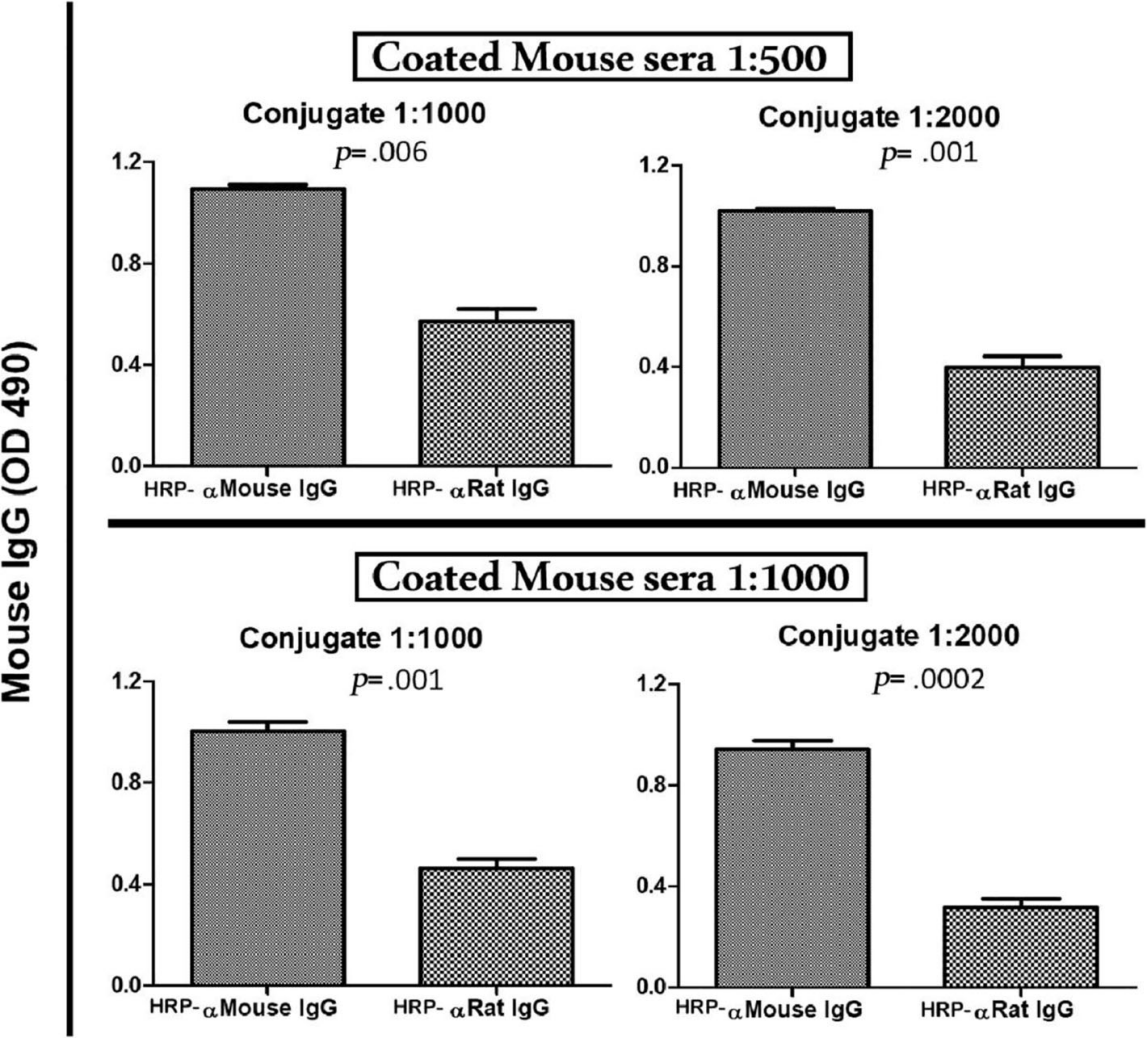
Reaction between mouse serum and either HRP-conjugated anti-mouse-IgG and HRP-conjugated anti-rat-IgG: Mouse sera (*n* = 5) were used at dilutions 1:500 and 1:1000 and the two conjugated antibodies were used at dilutions 1:1000 and 1:2000. Analysis of the obtained results using Students’ *t*-test showed statistically significant higher reactivity of the homologous antibody compared to a much less reactivity to the heterologous HRP-anti-rat-IgG antibody. The background cross-reaction of heterologous antibody further dropped upon increasing sera and conjugate dilutions (reaction with both homologous and heterologous antibodies were 0.9414±0.14 and 0.317 ±0.141, respectively (*p=* .0002) with a Δ mean O.D. of 0.624)

Direct ELISA assay was used to compare between HRP-conjugated antibody of both anti-mouse IgG (homologous) and anti-rat IgG (heterologous) for the detection of mouse sera IgG. The numerical results are shown in Table 1 for mouse serum coat section and plotted in Fig. 2. HRP-anti-rat-IgG reaction showed a low cross-reactivity in detecting mouse serum IgG. The dilution factor optimization started as mouse serum coating the plates at dilution of 1:500 reacting with both conjugated antibodies at dilution 1:1000. The reaction with both homologous and heterologous antibodies were 1.094±0.1 and 0.571±0.2, respectively (*p=* .006) with a Δ mean O.D. of 0.523. This difference was further improved by increasing the dilution factor of both mouse serum to 1:1000 and conjugate antibody to 1:2000. The later homologous and heterologous reactions were 0.9414±0.14 and 0.317 ±0.141, respectively (*p=* .0002) with a Δ mean O.D. of 0.624.

## Discussion

Sandwich ELISA detects an antigen trapped between two layers of antigen-specific antibodies originated from two different animal species. The bottom layer coating the reaction plate is considered the capturing antibody (Ab-c), while the upper layer serves as the detection antibody (Ab-d). The Ab-d is then detected by an enzyme-conjugated anti-species IgG-specific antibody. Actually, the conjugated antibody application allows it to interact with both the Ab-c and the Ab-d layers. The key step for confidence in the antigen detection depends on both the specificity and sensitivity of the conjugated antibody to detect the Ab-d layer.

The rationale of our study was to evaluate antibody detection specificity for a basic sandwich ELISA assays so the animals in our study were selected randomly and were treatment naïve. Our direct approach was to test the conjugate antibody for its possible interaction with the other species serum that will be used as Ab-c. In general, the degree of non-specific interaction of conjugated anti-mouse IgG antibody has been tested against rat serum and vice versa. This experiment allows lowering the possible cross-reaction by correcting the species orientation of the sandwich.

Our results showed that the coated rat serum IgG detection was nearly the same using the conjugate anti-IgG antibody of both anti-rat (homologous) and anti-mouse (heterologous). The difference between the respective mean O.D. reads was 0.14 using different concentrations of both coated rat serum and conjugate antibody. This low difference between the homologous and heterologous reactions reflects an unaccepted non-specific reaction of the conjugate anti-mouse IgG antibody. This means that its application will be misleading to an assay where rat serum is used as Ab-c.

On the other hand, the coated mouse serum IgG detection was different using the conjugate anti-IgG antibody of both anti-mouse (homologous) and anti-rat (heterologous). The difference between the respective mean O.D. reads was 0.522 in the highest concentration test reaction between 1:500 mouse serum coats and 1:1000 of both conjugate antibodies compared to each other. This difference can be improved to a value of 0.624 O.D. read using a higher dilution of coated mouse serum of 1:1000 and 1:2000 of conjugate antibody. This improved difference will allow the use of conjugate anti-rat IgG antibody to detect the rat Ab-d with a minimized cross-reaction with the coated mouse Ab-c.

Our results were supported by the report on a “striking cross reaction” between mouse IgG3 and rat IgG2c and not vice versa; this observation was associated with anti-carbohydrate specificity of the antibody [25]. No further experimental reports were found especially on a polyclonal raised antibody repertoire, which is commonly used in laboratory experiments.

The surprising difference in results with different species orientation amplifies the effect of the specificity bias. The diverse applications that depend on antibodies raised in laboratory animals should be subjected to specificity checkpoints. The described concept is simple yet directly improves other assays and aspects rather than that of the sandwich ELISA. The feasibility of antibody production and specificity optimization can also ease the problem of either the commercial unavailability of antigen-specific antibodies or the high cost of available specific antibody purchases. The delimited costs can be of a high importance especially for the low- and medium-income countries.

## Conclusions

Our results suggest that it is possible to use antibodies raised in both Swiss albino mice and albino rats in a single sandwich ELISA, given that the Ab-c to be from the Swiss albino mice and the Ab-d to be from the albino rat. This suggested species orientation will help in lowering non-specific cross-reaction and emphasize correct antigen detection/quantification. It also suggests that the species orientation test described herein is to be followed as a routine in sandwich ELISA assay optimization. The proof of the concept does not obligate the use of the suggested dilution factors that should be optimized for each experiment.

## Data Availability

Can be provided upon request.

## Abbreviations

ELISA: Enzyme-linked immunosorbent assay
IgG: Immunoglobulin G
HRP: Horseradish peroxidase enzyme label
O.D.: Optical density
PBS: Phosphate buffer saline
FBS: Fetal bovine serum
Ab-c: Capturing antibody
Ab-d: Detection antibody
